# Long Non-Coding RNAs Embedded in the Rb and p53 Pathways

**DOI:** 10.3390/cancers5041655

**Published:** 2013-12-04

**Authors:** Murugan Subramanian, Matthew F. Jones, Ashish Lal

**Affiliations:** Genetics Branch, National Cancer Institute, National Institutes of Health, Bethesda, MD 20892, USA; E-Mails: murugan.subramanian@nih.gov (M.S.); matthew.jones2@nih.gov (M.F.J.)

**Keywords:** cell cycle, lncRNA, miRNA, p53, Rb

## Abstract

In recent years, long non-coding RNAs (lncRNAs) have gained significant attention as a novel class of gene regulators. Although a small number of lncRNAs have been shown to regulate gene expression through diverse mechanisms including transcriptional regulation, mRNA splicing and translation, the physiological function and mechanism of action of the vast majority are not known. Profiling studies in cell lines and tumor samples have suggested a potential role of lncRNAs in cancer. Indeed, distinct lncRNAs have been shown to be embedded in the p53 and Rb networks, two of the major tumor suppressor pathways that control cell cycle progression and survival. Given the fact that inactivation of Rb and p53 is a hallmark of human cancer, in this review we discuss recent evidence on the function of lncRNAs in the Rb and p53 signaling pathways.

## 1. Introduction

The cell cycle is tightly regulated by complex molecular signaling pathways, but there are two important nodes that oversee many growth checkpoints. The retinoblastoma protein (pRb) and the p53 transcription factor are the two main tumor-suppressor pathways that control cellular responses to potentially oncogenic stimuli such as repeated cell division, DNA damage, and inappropriate mitogenic signals. Each pathway consists of several upstream regulators and downstream effectors, and both pathways lead first to cell cycle arrest and eventually to apoptosis or to senescence [1,2]. p53 mainly activates the transcription of a large number of genes in the context of DNA damage by directly binding to p53 recognition elements in the DNA close to or within the body of a target gene [3]. The downstream effects of p53 stem mainly from the induction of these myriad p53-target genes. However, p53 has also been shown to directly repress the expression of many genes [3,4,5]. These p53 activated genes include *CDKN1A* (also called p21), an inhibitor of cyclin-dependent kinases (CDKs) that causes cell cycle arrest, and BAX, which promotes apoptotic cell death [6]. In response to genotoxic stress, p53 can also carry out positive autoregulation by inducing expression of ARF (p14), which inhibits the p53-repressor MDM2 [7]. p53 can also engage in negative autoregulation by inducing p21/Cip1, genes encoding proapoptotic proteins such as *PUMA*, and *MDM2*, all of which play a role in terminating the p53 response [8]. 

**Figure 1:**
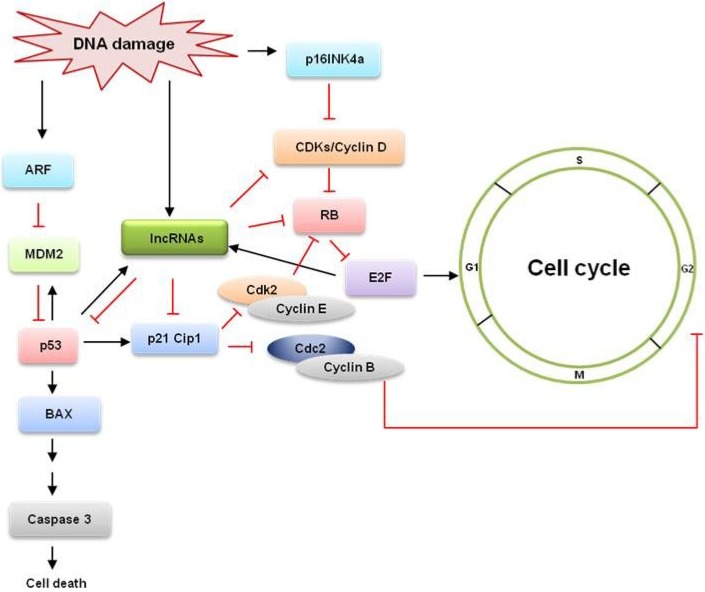
The tumor suppressor pathways p53 and retinoblastoma (RB) control the DNA damage response. p16INK4a and p14ARF controls the activity of RB and p53. RB promotes cell cycle arrest in G1 and regulates entry into S phase by inhibiting the E2Fs. p53 mediates several effects, including causing G1 and G2 arrest and promoting apoptosis. Loss of p53 function also promotes genomic instability. Several lncRNAs regulate the expression of cyclins-CDKs, CKIs, pRB, E2F and p53 and are thereby involved in the regulation of cell cycle. Some of these lncRNAs are induced by DNA damage and inhibit cell cycle progression.

The pRb pathway supervises normal cell cycle progression as well as stress responses. Cell cycle progression is directly controlled by a series of CDKs that bind to their respective cyclins and are subsequently phosphorylated by an activating kinase [9]. The cycle starts in G1 with elevated levels of the D cyclins (D1, D2, and D3) which activate CDK4 and CDK6 [10]. The activated Cyclin D-CDK4/6 complex phosphorylates pRb and the related proteins p107 and p130, which are also important for regulating E2F activity [1]. In its hypophosphorylated state, pRb forms a stable complex with E2F1, preventing it from activating the transcription of cell cycle genes; phosphorylation by CDK4/6 causes dissociation of the pRb/E2F complex, and liberation of E2F1 is necessary for the transcription of Cyclins E and A (Figure 1). Production of cyclins E and A are required for progression through the G1 and S phases of the cell cycle [11]. In pRb-mediated growth arrest, stress signals induce p16INK4a, which specifically binds and inhibits CDK4/6, thus preventing the phosphorylation and inactivation of pRb during G1 [12]. In addition to sequestering E2F1, hypophosphorylated pRb also controls the expression of hundreds of genes by recruiting transcription factors and chromatin remodeling proteins to E2F-responsive promoters [11]. 

The p53 and pRb pathways intersect with the formation of a trimeric p53-MDM2-Rb complex. The binding of Rb to MDM2 is required for Rb to overcome both the antiapoptotic function of MDM2 and the MDM2-dependent inactivation of p53 [13]. In addition to functional links between the p53 and Rb tumor suppressor networks at the protein level, many studies have presented evidence for targeting of mRNAs that encode proteins directly or indirectly involved in cellular proliferation by microRNAs (miRNAs) [14]. MicroRNAs are ~22 nucleotide (nt) small non-coding RNAs which mediate sequence specific, post-transcriptional repression of mRNA targets [15]. For example, the miR-15a-16-1 cluster induces cell cycle arrest at G1 phase by targeting critical cell cycle regulators such as CDK1, CDK2 and CDK6 as well as cyclins D1, D3 and E1 [16,17]. CDK4 or CDK6 mRNAs are also targeted by other miRNAs, including miR-24, miR-34a, miR-124, miR-125b, miR-129, miR-137, miR-195, miR-449 and the let-7 family members [18,19,20,21,22]. Many other miRNAs have also been reported to regulate key players in cell cycle control, and many miRNAs display antiproliferative properties and function as tumor suppressors [14,23]. 

Recent advances in high-throughput functional genomics have led to the identification of a new type of regulatory RNAs called long non-coding RNAs (lncRNAs) [24]. LncRNAs are >200 nt long, generally poorly conserved, and they regulate gene expression by diverse mechanisms that are not yet fully understood [25,26,27]. Based on structural characteristics, lncRNAs can be classified into different categories including antisense, intronic, intergenic, pseudogenes, and retrotransposons. Emerging evidence suggests that lncRNAs act via diverse mechanisms such as epigenetic regulation, transcriptional control, post-transcriptional regulation and molecular scaffolding [28]. From this standpoint, lncRNAs are quite different from miRNAs because miRNAs mainly inhibit gene expression at the post-transcriptional level. Although only a small number of lncRNAs have been well-characterized, distinct lncRNAs have been shown to play a role in X-chromosome inactivation, genomic imprinting, sub-cellular structural organization, telomere and centromere organization and nuclear trafficking [28,29,30,31,32]. Moreover, the expression of lncRNAs is dysregulated in a number of human diseases, including coronary artery diseases, neurological disorders, autoimmune diseases, and cancer [33,34,35]. Furthermore, there is a growing amount of evidence for crosstalk between miRNA and lncRNA pathways. The lncRNA PTENP1 binds PTEN-inhibitory miRNAs and limits their activity [33,36]; transcribed ultraconserved regions are transcriptionally regulated by miRNAs [36]; and transcriptome-wide PAR-CLIP analysis has recently revealed widespread association of miRNA-Argonaute complexes with lncRNA [37].

In this review, we have focused on lncRNAs involved in regulating the cell cycle, particularly via the p53 and Rb pathways. Some lncRNAs have recently been implicated in regulating the expression of several cell cycle genes, many of which relate to p53 signaling due to the central role of p53 in cell cycle control and apoptosis. We discuss some of the recent findings on lncRNAs in the pRb and p53 pathways lncRNAs below (Table 1). 

**Table 1:** lncRNAs involved in the p53 and RB pathway.

lncRNA	Targets and mechanism of action	References
lncRNA-p21	Inhibition of transcription of target genes involved in apoptosis and cell cycle through hnRNPK; also represses the translation of β-catenin and Jun B mRNA translation through HuR	[38,39,40]
PANDA	Interacts with the transcription factor NF-YA to limit expression of proapoptotic genes	[41]
MEG3	Interacts with p53 to transactive target gene promoters	[42,43,44,45,46]
LincRNA-RoR	Inhibits translation of p53 mRNA	[47,48]
LOC285194	Inhibits function of miR-211	[49]
MALAT1	Down-regulates p53 and target genes; also up-regulates pro-metastatic genes such as MIA2 and ROBO1	[50,51,52,53,54]
p53-induced eRNAs	Promotes p53 target gene transcription	[55]
H19	Down-regulates RB mRNA translation through miR-675	[56]
KCNQ1OT1	Inhibits p57 transcription	[57,58,59,60,61,62,63]
ANRIL	Represses the INK4b-ARF-INK4a locus with PRC1/2	[64,65,66,67,68]
HULC	Sequestration of miR-372, causing derepression of PRKACB; also suppress p18 expression	[69,70]
ncRNA_CCNDI_	Represses CCND1 transcription with activation of TLS	[71]
GADD7	Inhibits TDP43 by sequestration of TDP43	[72,73]

## 2. lncRNAs Involved in the p53 Pathway

The p53 pathway is well studied in the context of regulating cell cycle and apoptosis and maintaining genomic integrity. Therefore, it is an attractive starting point for the investigation of new molecular components involved in these processes. MiRNAs have previously been reported as important regulatory partners of p53 [74], but emerging evidence suggests that other types of non-coding RNAs are also involved. Below, we discuss the roles of long non-coding transcripts in p53 signaling.

### 2.1. Long Non-Coding RNAs at the CDKN1A (p21) Locus—LincRNA-p21

**Figure 2:**
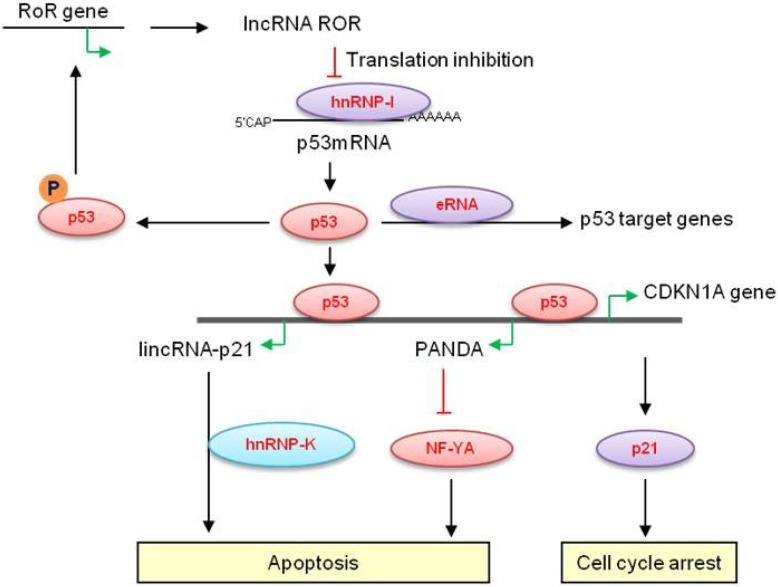
Proposed model for the role of lncRNAs in p53 pathway. LncRNA-RoR binds to hnRNP-I to suppress p53 mRNA translation and phosphorylated p53 activates the lncRNA-RoR transcription. This forms an autoregulatory feedback loop that controls p53 levels. After DNA damage p53 directly binds at the CDKN1A locus and activates the transcription of *CDKN1A*, *PANDA* and LincRNA-p21. p21 mediates cell cycle arrest, PANDA inhibits NF-YA to block apoptosis and LincRNA-p21 mediates gene silencing through recruitment of hnRPK to promote apoptosis. p53 induced eRNAs are involved in activation of p53-target genes and mediate p53-dependent cell cycle arrest.

Although p53 can activate or repress gene expression, the mechanisms by which p53 mediates gene repression are less well-understood. Recently, Huarte *et al*. reported that many lincRNAs are physically associated with repressive, chromatin-modifying complexes and suggested that they may serve as repressors in p53 transcriptional regulatory networks. Using custom tiling microarrays representing 400 lncRNAs they identified several lncRNAs up-regulated after DNA damage in mouse embryonic fibroblasts (MEFs) and in a mouse lung tumor cell line. The authors demonstrated a role for a long intergenic lncRNA (lincRNA) lincRNA-p21 in the p53 pathway. LincRNA-p21 is transcribed from a locus 15 kb upstream of *CDKN1A* (p21) on the negative strand. The lincRNA-p21 promoter contains a consensus p53 motif to which p53 binds directly, and its transcription is up-regulated upon DNA damage in MEFs [38]. Depletion of lincRNA-p21 by siRNAs alters the expression of several p53 target genes, with the notable exception of p21, to induce apoptosis in MEFs and in the mouse lung tumor cell line. Upon activation, lincRNA-p21 binds to the heterogeneous nuclear ribonucleoprotein K (hnRNPK) and recruits it to repressive transcriptional complexes to assist p53 in transcriptional repression (Figure 2) [38]. Furthermore, this study identified a highly conserved region of 780 nucleotides at the 5' end of lincRNA-p21 necessary for hnRNPK binding and for the induction of apoptosis in cells exposed to DNA damage. With respect to conservation, an orthologous 5′exon region of human lincRNA-p21 was found to be strongly induced by p53 in human fibroblasts. 

Following this initial report, Yoon *et al*. proposed a new role for lincRNA-p21 as a post-transcriptional inhibitor of translation [39]. Through the use of pulldown techniques, the authors demonstrated an association between the RNA binding protein HuR and lincRNA-p21, which led to the recruitment of the miRNA let-7b and Ago2 to regulate the half-life of lincRNA-p21. Furthermore, they showed that lincRNA-p21 contains many regions complementary to *CTNNB1* (β-catenin) and *JUNB* mRNAs, which encode proteins involved in promoting cell survival and proliferation. These mRNAs were translationally repressed upon HuR depletion [39]. This study suggested that HuR is important for the post-transcriptional regulation of *CTNNB1* and *JUNB* mRNAs via the degradation of lincRNA-p21 [39]. Therefore, these studies suggest that lincRNA-p21 regulates transcription in the nucleus and translation in the cytoplasm. 

In a recent study, Zhai *et al*. investigated the impact of lincRNA-p21 in colon cancer progression. Consistent with the aforementioned results in MEFs and mouse lung cancer cells, lincRNA-p21 levels were up-regulated upon activation of p53 in CRC cell lines. However, they did not observe any correlation between lincRNA-p21 and p53 mutation status in colorectal cancer [40]. 

### 2.2. p21-Associated ncRNA DNA Damage-Activated—PANDA

Using high-density tiling arrays Hung *et al*. profiled the transcription and chromatin landscape at the promoters of 56 cell cycle genes [41]. They found hundreds of long RNAs expressed from cell cycle promoters, distinct from previously described transcription start site associated RNAs (TSS-RNAs) [75] in their greater length and distance from transcription start sites (TSSs). Several lncRNAs transcribed from the *CDKN1A* (p21) promoter were found to be rapidly up-regulated after doxorubicin-induced DNA damage. In particular, one lncRNA termed *PANDA*, was characterized as a 5′-capped, polyadenylated, monoexonic p53 target RNA (Figure 2). *PANDA* knockdown sensitized fibroblasts to apoptosis following DNA damage by interfering with NF-YA occupancy of the core promoter regions upstream of p53-targeted cell death genes including *CCNB1*, *FAS*, *PUMA*, and *NOXA* [41]. Interestingly, a gain-of-function p53 mutation (R273H) associated with Li Fraumeni syndrome [76] abolished p21 induction but retained the ability to induce *PANDA* expression. In a subsequent study interrogating lncRNA expression in HeLa and *CASP3*^−/−^ MCF7 cells following bleomycin- or γ-radiation-induced DNA damage, no significant differences in *PANDA* expression were observed [77]. Therefore, factors other than p53 also play a role in the regulation of *PANDA* expression, and *PANDA* may represent a highly context-dependent response to certain types of genotoxic stress. 

### 2.3. MEG3

Whereas *PANDA* is a p53-responsive lncRNA, another lncRNA *MEG3* has been shown to exert potent anti-proliferative effects by regulating p53 itself. *MEG3* was the first lncRNA to be attributed tumor suppressive function [78]. It was first described as a paternally imprinted locus encoding a 10-exon RNA [42], and in a notable similarity to the likewise monoallelically expressed H19 (see below), miR-770 derives from the final exon of *MEG3* [43]. Due to the loss of *MEG3* expression in pituitary tumors compared to normal tissue, Zhang *et al*. first proposed that MEG3 acts as a tumor suppressor, and this observation was supported by the finding that *MEG3* reintroduction into cancer cell lines inhibits proliferation [44]. This initial conception of *MEG3* function can be mechanistically explained by its interactions with p53, whereby it facilitates p53 binding to and transactivation of target gene promoters [45]. Crucially, this interaction with p53 depends exclusively on the secondary structure of the *MEG3* transcript, as replacing *MEG3* exons with different sequences does not affect p53 activation or growth suppression provided the secondary structure is preserved [46]. Not only does this sequence independence support arguments for the compatibility of rapid evolution and functional relevance with regards to lncRNA [77,79], but it also indicates that *MEG3* is unlikely to carry out its p53-independent growth suppressive functions [45] via a sequence-specific mechanism such as targeting chromatin remodeling enzymes to specific genomic regions. 

### 2.4. LincRNA-RoR

Three overlapping lincRNAs: lincRNA-SFMBT2, lincRNA-VLDLR and lincRNA-ST8SIA3 (named lincRNA-RoR), were recently identified by Loewer, *et al*. These lincRNAs were up-regulated during somatic cell reprogramming to induced pluripotent stem cells (iPSCs). Silencing or over-expression of lincRNA-RoR correlated directly with iPSC colony formation. Colocalization of Oct4, Sox2, and Nanog near the lincRNA-RoR promoter region indicates that its expression is induced by the key pluripotency factors [47]. Later, Zhang, *et al*. expanded on this result by reporting that p53 expression can be negatively regulated by lincRNA-RoR, thereby mediating cell cycle arrest and apoptosis. Silencing lincRNA-RoR leads to p53 accumulation, and enforced expression of lincRNA-RoR suppresses p53 induction after doxorubicin treatment. LincRNA-RoR binds to heterogeneous nuclear ribonucleoprotein I (hnRNPI) and this interaction occurs in the cytoplasm where phosphorylated hnRNPI is predominantly localized (p-hnRNPI) (Figure 2). LincRNA-RoR interacts with p-hnRNPI to mediate p53 repression through translational regulation, and this suppression was especially magnified in the context of DNA damage [48]. Furthermore, this study identified a 28-base RoR sequence carrying the potential hnRNP I binding motifs that is both necessary and sufficient for p53 repression [48].

### 2.5. LOC285194

LncRNA-LOC285194 is a 2 kb long RNA located at chr3q13.31 which harbors frequent focal copy number alterations (CNAs) and loss of heterozygosity (LOH) in primary osteosarcoma samples as well as in cell lines from other malignancies. This so-called osteo3q13.31 region includes the lncRNAs LOC285194 and BC040587, which were lost in osteosarcoma. The loss of these lncRNAs in cancer suggested tumor suppressor roles, yet the functional mechanism was unknown [80]. Recently, Liu *et al.* found that LOC285194 is activated by p53 in response to DNA damage by doxorubicin. Ectopic expression of LOC285194 inhibits tumor cell growth whereas silencing LOC285194 increases cell growth in colon cancer cell lines. This study also found that LOC285194 contains binding sites for miR-211 and thereby sequesters and inhibits miR-211, resulting in decreased cell proliferation. Finally, this study identified a clinical correlation that LOC285194 is down-regulated in human colon cancer tumors compared to normal tissue [49], suggesting a potential role in colorectal cancer.

### 2.6. MALAT1

MALAT1 was first identified as a well-conserved, prognosis-related RNA in lung cancer, where its expression varied inversely with metastasis-free survival [81]. However, its function remained uncharacterized for several years. Screens for factors involved in RNA splicing subsequently revealed a role for MALAT1 (a.k.a. NEAT2) in mRNA splicing [82]. Although MALAT1 was originally described as regulating alternative splicing in nuclear speckle subdomains by modulating localization of phosphorylated SR-splicing factors [50], we have recently connected MALAT1 to cell cycle control via regulation of p53 levels [51]. We showed that MALAT1 is necessary for normal cell cycle progression, particularly the transition from G2 to M. This effect is mediated by (a) down-regulation of p53 and its target genes, and (b) up-regulation of B-MYB expression by MALAT1 [51]. B-MYB, in turn, promotes the transcription of cell cycle progression genes, including FOXM1, CDK1, and cyclin B1. These functional studies provide valuable insights into the oncogenic functions previously attributed to MALAT1. However, further studies will be needed to understand exactly how MALAT1 regulates the expression of p53, whether by its previously described alternative-splicing activity or a yet-unknown mechanism. A MALAT1 knockout model in lung cancer cell lines has shown a direct relationship between MALAT1 and increased metastasis. Contrary to the known effect of MALAT1 on alternative splicing, Gutschner *et al.* demonstrated that MALAT1 promotes apoptosis by up-regulating the expression of pro-metastasis genes such as *MIA2* and *ROBO1* [52].

Interestingly, embryonic mouse knockout models for MALAT1 displayed no discernible phenotype. mRNA transcripts were spliced at normal levels and nuclear speckles were correctly formed and localized [53]. The authors speculated that this was due to cell-type specific effects of MALAT1, but a subsequent study found that, although MALAT1 knockout mice developed normally, adults displayed consistent alterations in the expression of MALAT1-adjacent genes [54]. Therefore, MALAT1 may also act in *cis* to regulate the expression of its neighbors. Whether this mechanism is germane to the effects of MALAT1 on metastasis, cell cycle, and p53 remains to be seen. 

### 2.7. P53-Induced Enhancer RNAs

In a recent study, Melo *et al*. reported a novel mechanism by which p53 enhances gene expression via transcription of a class of non-coding RNAs called enhancer RNAs (eRNAs) [55]. This study analyzed genome-wide p53 binding profile via ChIP-seq to identify novel p53 transcriptional targets. They demonstrated that p53 binds to and promotes the transcription of enhancer regions termed p53-bound enhancer regions (p53BER) (Figure 2). Furthermore, by using chromosome conformation capture combined with next-generation sequencing they showed that p53BERs interact intrachromosomally with multiple neighboring genes. Silencing the eRNAs that are produced at the p53BERs by siRNAs inhibited p53-dependent cell cycle arrest and these eRNAs are required for p53-dependent long-distance activation of promoters [55]. Their studies therefore provide further evidence on the role of non-coding regions of the genome in p53 signaling. 

## 3. LncRNAs Involved in the Rb Pathway

In addition to their intersection with p53-mediated cell cycle control, a number of lncRNAs have been implicated in another tumor-suppressor network, namely that of pRB. This is brought about, in part, by lncRNA regulation of cyclins and cyclin-dependent kinases (CDKs). 

### 3.1. H19

The H19/IGF2 at chromosome 11p15.5 locus encodes both the IGF2 secreted protein and a 2.9 kb lncRNA H19, the expression of which is controlled by the target of pRB inhibition E2F1. H19 lies within 200 kb of *IGF2*, but it has long been known that H19 and its neighbor IGF2 are reciprocally imprinted, such that only the maternal *IGF2* allele is expressed while H19 is only expressed from the paternal allele [83,84]. The H19 transcript is a precursor for miR-675, which is associated with a decreased proliferatory phenotype [56]. The lncRNA H19 itself appears to promote entry to S-phase downstream of E2F1, resulting in increased growth rate and tumorigenesis. LncRNA H19 is most abundant in embryonic tissues, but its expression is reactivated in several types of human cancer, including breast, bladder [85], and gastric carcinomas [86], where its expression is negatively correlated with the cyclin-dependent kinase inhibitor (CDKi) p57^kip2^ [87,88]. 

**Figure 3:**
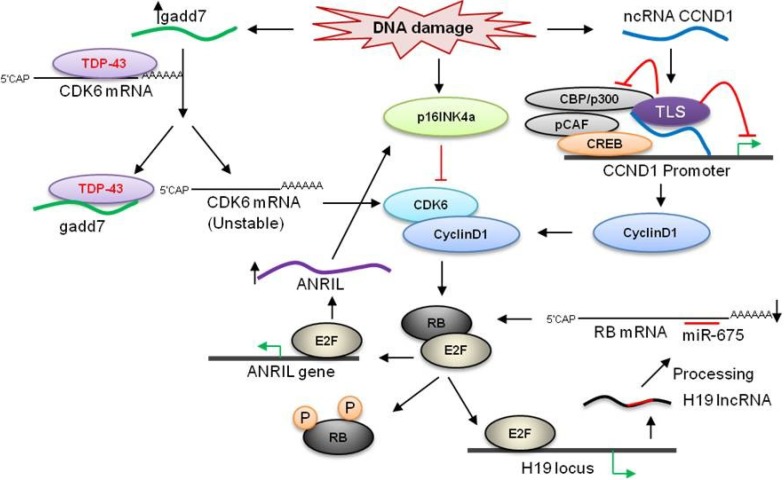
Proposed model for the role of lncRNAs in RB pathway. Cyclins and CDKs are key players in cell cycle regulation. The promoter associated non-coding RNA CCND1 (ncRNA CCND1) functions as a transcription factor regulator. NcRNA CCND1 is induced upon DNA damage and negatively regulates the expression of Cyclin D1. The ncRNA CCND1 along with TLS inhibits the coactivator, CBP/p300 to inhibit the transcription of CCND1 in its promoter. The lncRNA gadd7 induced by DNA damage destabilizes the CDK6 mRNA by interacting with TDP-43. TDP-43 is important for stability of CDK6 mRNA. CDK4/6-Cyclin Ds phosphorylates the RB and activates E2F which regulates the expression of several proteins that promote the S phase entry. H19 lncRNA transcribed by E2F from H19 locus is processed to yield miR-675, which regulates the translation of RB mRNA. E2F1 transcriptionally activates ANRIL, which consequently represses the expression of p16INK4 family members, and thus alleviates p53 and pRB signaling pathways in response to the DNA damage. ANRIL repress p16INK4 by interaction with PRC2 complex at the INK4 locus.

Intriguingly, a natural antisense transcript of H19, termed 91H, has also been detected in breast cancer, and is associated with increased expression of *IGF2* on the paternal allele in *trans* [89]. Furthermore, the H19-derived miR-675 may also play a role in negative feedback with pRB by directly inhibiting the *RB1-*3'UTR (Figure 3) [90]. H19 has also been shown to be directly activated by oncogenic c-Myc, which is consistent with a pro-growth model of H19 function. However, despite molecular evidence for oncogenic H19 functions in tissue culture systems, tumor suppressive functions have been attributed to H19 *in vivo* [91]: a hepatocarcinoma mouse model with an H19 knockout developed tumors much earlier than wild-type, a similar model for teratocarcinoma showed decreased tumor growth compared to wild-type embryos, and *APC*Δ14*/+* mice with intact H19 developed fewer polyps than H19 knockouts [92]. Given the reported inhibition of IGF1R by miR-675, there is a complex interplay between the suite of ncRNAs located at H19, the pRB pathway, and IGF signaling, and further work will be necessary to clarify the mechanisms by which these RNAs promote cell proliferation and tumorigenesis. It may be the case that the expression of the ncRNAs at the *H19* locus- miR-675, H19, and 91H- is highly context-dependent and can exert varying effects in different molecular environments. Matters are further complicated by the recent discovery of a primate-specific, tumor suppressive protein encoded antisense to H19, so-called *HOTS*, the undetected presence of which may have confounded earlier H19/91H loss-of-function studies [93].

### 3.2. KCNQ1OT1

Like *H19*, *KNCQ1OT1* is also located at 11p15.5, paternally imprinted, and its over-expression is associated with cancer progression, increased proliferation, and the overgrowth disorder Beckwith-Wiedemann Syndrome (BWS) via the inhibition of p57 expression [57,58]. Repression of eight protein coding genes, including p57, in a 1 MB region around *KCNQ1OT1* depends on *KCNQ1OT1* expression [59]. Pandey *et al*. demonstrated that *KCNQ1OT1* mediates bidirectional epigenetic silencing of its neighboring genes on the paternal chromosome by associating with histone methyltransferases and PRC2 [60]. Over-expression of *KCNQ1OT1* is a feature of breast [61], colon [62], and hepatocellular carcinoma tumors and cell lines [63]. It is unclear whether *H19* and *KCNQ1OT1*-mediated regulation of their respective imprinted gene clusters on chromosome 11 participate in any significant crosstalk, but their proximity and pathophysiological similarities are striking examples of the importance of lncRNAs in epigenetic modification.

### 3.3. ANRIL

While H19 mediates the downstream effects of the retinoblastoma protein, the stability of pRB itself is tightly controlled by a well-characterized pathway involving the *INK4A/ARF/INK4B* genes and *CDK4/6* [94,95,96]. Briefly, the INK4 gene cluster contains genes *INK4A*, *INK4B*, and *ARF* (also known as *CDKN2A*, *CDKN2B*, and *INK4A^ARF^*) coding for the proteins p15, p16, and ARF, which inhibit CDK4 and 6, respectively, thereby preventing phosphorylation and inactivation pRB by CDK4/6. The alternative-reading frame-encoded ARF intersects with both p53 and pRB by inhibiting their MDM2-dependent degradation [97]. The expression of the genes at the *INK4* locus is, in turn, controlled by methylation at histone 3, lysine 27 (H3K27me) in regulatory regions by the polycomb repressive complex (PRC) [98]. Despite the decades of research into regulation of pRB, it is only now becoming apparent that lncRNAs are critical for the precisely timed activation and inactivation of pRB by guiding the PRC to the INK4 locus and that dysregulation of lncRNA expression may contribute to loss of pRB in cancer [99]. The lincRNA *ANRIL* is a 3.8 kb, multi-exonic transcript that spans over 120 kb of the genome. ANRIL overlaps the p15 portion of the INK4 gene cluster, but is transcribed in the antisense direction [64]. The first mechanistic study of *ANRIL* suggested that ANRIL recruits PRC proteins in *cis* to silence the expression of the *INK4A/ARF* locus, but not *INK4B* [65]. Kotake, *et al* later called these results into question with their report that deposition of H3K27me by SUZ12, a PRC2 component, at the *p15/INK4B* promoter was dependent on *ANRIL* expression. Loss or inhibition of *ANRIL* limits cellular lifespan and promotes senescence [66]. *ANRIL* is transcriptionally induced in the context of DNA damage by E2F1 downstream of ATM-ATR activation, where *ANRIL* caused inhibition of p15, p16, and, to a lesser extent, p14 (Figure 3). Induction of *ANRIL* by E2F1 was demonstrated by both luciferase-reporter assays and E2F1 chromatin immunoprecipitation (ChIP). It should also be noted that induction of *ANRIL* expression was observed in response to DNA damage in cell lines treated with p53 shRNA and p53^−/−^ cell lines. Therefore *ANRIL* expression is controlled in a p53-independent manner [34,35,67]. As *ANRIL* over-expression resulted in decreased rates of apoptosis and cell cycle arrest, the authors proposed a role for *ANRIL* in relieving the DNA-damaged cell of p53/pRB associated cell cycle control in the late phases of the DNA-damage response, thereby promoting homologous recombination and cell-cycle reentry [67]. These functional studies indicate the importance of *ANRIL* in the fine control of cell cycle and DNA damage response and point toward a feed-forward mechanism by which pRB inactivation can be maintained by E2F1 induction of *ANRIL*, leading to INK4 cluster silencing. 

Further analysis of *ANRIL* structure has revealed broader *trans-*regulatory capabilities consistent with its associated phenotypes of proliferation, adhesion, and decreased apoptosis. The *Alu* elements embedded in the spliced *ANRIL* transcript facilitate targeting of other *Alu* motifs across the genome, resulting in an enrichment of *ANRIL-*associated PRC proteins and concomitantly decreased expression at genes outside of the *INK4* cluster [68]. Seen in this light, early reports of GWAS hotspots the *ANRIL* region for breast cancer [100], basal cell carcinoma [101], nasopharyngeal carcinoma [102], glioma [103], coronary disease, aneurysm, and type 2 diabetes [104] may be less surprising than originally thought. 

### 3.4. HULC

Another member of the CDKi family, p18^INK4c^, has recently been shown to undergo lncRNA-mediated silencing in human cancer. The lncRNA *Highly Upregulated in Liver Cancer* (HULC), as its name would suggest, is overexpressed in hepatocellular carcinoma, where it is associated with *cis-*acting repression of p18 transcription [69]. HULC itself is controlled by cAMP signaling as part of an intricate auto-regulatory loop: CREB promotes *HULC* transcription, *HULC* in turn acts to “sponge” endogenous miRNAs such as miR-372, causing derepression of *PRKACB* and increased protein kinase A activity, eventually resulting in increased CREB phosphorylation and activity [70]. Further study will be needed to determine the mechanistic nature of the *HULC-INK4C* interaction, but it stands to reason that by integrating pro-growth signals from the cAMP-PKA pathway to silence a happloinsufficient tumor suppressor [105], *HULC* expression promotes proliferation, resistance to apoptosis, tumorigenicity, and metastasis [69]. 

### 3.5. ncRNA_CCND1_

The control of E2F1 activity by pRB regulates and is regulated by cyclins and the CDKs with which they cooperate. Cyclin D1 (CCND1) is an example of a cell cycle gene whose expression is governed by lncRNA dependent mechanisms. The discovery of lncRNAs regulating *CCND1* began with a screen to detect cellular components that modulate the activity of histone acetyl transferase (HAT) transcriptional coactivators. In a series of elegant experiments, Wang, *et al*. demonstrated that the protein TLS can inhibit the CBP/p300-dependent expression of CCND1 by binding a specific RNA motif. These motifs are present in ncRNAs transcribed from multiple regions upstream of the CCND1 locus after DNA damage. One of these transcripts, the low-abundance ncRNA*_CCND1_*, ligates and allosterically activates TLS, thereby inhibiting histone acetylation, CCND1 transcription, and G1-S cell cycle progression in response to genotoxic stress (Figure 3) [71].

### 3.6. Gadd7

Gadd7 was one of the first lncRNAs to be described in relation to the cell cycle. When it was first identified by Fornace *et al*. in 1988, it was something of a mystery that this transcript that inhibited proliferation when over-expressed did not appear to code for any proteins [72,106]. The expression of Gadd7 is increased by several different types of DNA damaging agents [73], indicating that it is an integral part of the DNA-damage response pathway, but it is still unclear what transcription factors control the upstream regulation of Gadd7. A recent result has clarified this matter by demonstrating a binding interaction between Gadd7 and TAR-binding protein TDP-43. This interaction prevents TDP-43 from binding to the CDK6 mRNA, resulting in destabilization and degradation of CDK6 mRNA (Figure 3). This mechanism not only explains previous functional observations [107], but it also suggests that Gadd7 and other lncRNAs may regulate other cell cycle genes at the post-transcriptional level.

## 4. Perspectives and Conclusions

We have presented an overview of interactions between lncRNAs and cell-cycle regulatory genes (Table 1). The study of lncRNAs is still very much in its infancy, and many of the results described above invite further investigations. Unlike miRNAs, around whose mechanism of action a consensus is rapidly emerging, it seems unlikely that lncRNAs exert their effects through shared molecular mechanisms. Rather, lncRNAs may represent an evolutionarily young [79] mode of gene regulation that operates via a plenitude of as-yet poorly understood pathways. Compelling evidence exists for the action of several lncRNAs in *cis*. Beyond the classical example of *XSIST* [108], several cell cycle lncRNAs including *PANDA*, *ANRIL*, *KCNQ1OT1*, *ncRNA_CCND1_*, exert their effects in a chromosome-of-origin specific manner. Other lncRNAs have been detected in the cytoplasm, where they may alter translation directly (lincRNA-RoR) or by modulating miRNA availability (HULC). A prominent theory of global lncRNA function involves competition between endogenous transcripts for free miRNA molecules [109], but this so-called ceRNA hypothesis seems to play only a minor part in cell cycle regulation. Still other lincRNAs have been shown to act in *trans*, such as *H19* and *PANDA*.

Moreover, there seems to be little concurrence between function and mechanism in the lncRNA world. That is, even a set of lncRNAs with similar functions will employ a diverse range of mechanisms from recruiting chromatin-remodeling enzymes to tethering transcription factors to influencing the sub-nuclear organization and localization of chromatin. Not only do the mechanisms of individual lncRNAs demand extensive characterization, there is also a lack of understanding of the position of lncRNAs within the broader gene-regulatory network of the cell. Do all lncRNAs operate according to *sui generis* mechanisms, or can any coherent pattern be observed? Does the lack of conservation in lncRNAs and the inclusion of “junk” DNA such as retrotransposons indicate functional irrelevance? How can lncRNAs interact with other non-coding transcripts, and to what extent do kinetics and stoichiometry govern effects that are normally measured using cruder, binary gain- and loss-of function assays? 

These are vital questions for the field to address, because existing data on lncRNAs indicates a real albeit distant promise for new genetic therapies. This review presents compelling evidence that lncRNAs are essential components of cell cycle control and comprise a novel layer of regulatory complexity. Dysregulation of lncRNA expression is associated with a broad range of human diseases including cancers, and lncRNAs offers several advantages as targets for clinical therapy. The tissue-specific expression patterns of lncRNAs are distinct from the ubiquitous expression of many miRNAs and protein-coding genes across multiple tissue types. Given this specificity, lncRNAs may be better biomarkers than miRNAs or mRNAs. For example, lncRNA HOTAIR [33,110], PCA3 [36,111] and UCA1 [37,112] have been treated as potential biomarkers of hepatocellular carcinoma recurrence, prostate cancer aggressiveness and bladder cancer diagnosis, respectively. Another major lncRNA in cancer is the metastasis-associated lung adenocarcinoma transcript 1 (MALAT1). Apart from lung adenocarcinoma, MALAT1 was implicated in hepatocellular carcinoma (HCC) [113]. Because lncRNAs can be specifically and efficiently inhibited using simple antisense chemistry, they make for attractive therapeutic targets [114,115]. In cancer, for example, significant progress is being made with miRNA-based therapies [116,117,118,119,120], and a greater understanding of lncRNAs will be a valuable addition to the growing therapeutic arsenal derived from RNA-mediated gene regulation. Therefore, identifying disease-related lncRNAs can facilitate not only the understanding of molecular mechanisms of cancer at lncRNA level, but also identification of biomarker for cancer diagnosis, treatment, prognosis and prevention.

Currently, nucleic acid-based methods provide powerful options for targeting RNA, either by regulating the level of lncRNAs in cancer cells or modifying their structures or mature sequences [121]. Employing RNA interference (RNAi) based techniques could be adapted to inhibit the lncRNAs function in cancer cells [122]. Both siRNAs and shRNAs exhibit high knockdown efficiency for lncRNAs and the simplicity of siRNAs and shRNAs synthesis makes them attractive as versatile therapeutic agents. Other established methods such as antisense oligonucleotide (ASO), ribozymes and aptamers, are also effective modulators of lncRNA expression. The development and use of multiple approaches is important to identify the most effective lncRNA-based therapy. 
